# Visualisation of *Leishmania donovani* Fluorescent Hybrids during Early Stage Development in the Sand Fly Vector

**DOI:** 10.1371/journal.pone.0019851

**Published:** 2011-05-27

**Authors:** Jovana Sadlova, Matthew Yeo, Veronika Seblova, Michael D. Lewis, Isabel Mauricio, Petr Volf, Michael A. Miles

**Affiliations:** 1 Department of Parasitology, Faculty of Science, Charles University, Prague, Czech Republic; 2 Department of Pathogen Molecular Biology, Faculty of Infectious and Tropical Diseases, London School of Hygiene and Tropical Medicine, London, United Kingdom; 3 Instututo de Higiene e Medicina Tropical, Lisboa, Portugal; The University of Maryland, United States of America

## Abstract

**Background:**

The *Leishmania* protozoan parasites cause devastating human diseases. *Leishmania* have been considered to replicate clonally, without genetic exchange. However, an accumulation of evidence indicates that there are inter-specific and intra-specific hybrids among natural populations. The first and so far only experimental proof of genetic exchange was obtained in 2009 when double drug resistant *Leishmania major* hybrids were produced by co-infecting sand flies with two strains carrying different drug resistance markers. However, the location and timing of hybridisation events in sand flies has not been described.

**Methodology/Principal Findings:**

Here we have co-infected *Phlebotomus perniciosus* and *Lutzomyia longipalpis* with transgenic promastigotes of *Leishmania donovani* strains carrying hygromycin or neomycin resistance genes and red or green fluorescent markers. Fed females were dissected at different times post bloodmeal (PBM) and examined by fluorescent microscopy or fluorescent activated cell sorting (FACS) followed by confocal microscopy. In mixed infections strains LEM3804 and Gebre-1 reached the cardia and stomodeal valves more rapidly than strains LEM4265 and LV9. Hybrids unequivocally expressing both red and green fluorescence were seen in single flies of both vectors tested, co-infected with LEM4265 and Gebre-1. The hybrids were present as short (procyclic) promastigotes 2 days PBM in the semi-digested blood in the endoperitrophic space. Recovery of a clearly co-expressing hybrid was also achieved by FACS. However, hybrids could not sustain growth in vitro.

**Conclusions/Significance:**

For the first time, we observed *L. donovani* hybrids in the sand fly vector, 2 days PBM and described the morphological stages involved. Fluorescence microscopy in combination with FACS allows visualisation and recovery of the progeny of experimental crosses but on this occasion the hybrids were not viable in vitro. Nevertheless, genetic exchange in *L. donovani* has profound epidemiological significance, because it facilitates the emergence and spread of new phenotypic traits.

## Introduction

Protozoan parasites of the genus *Leishmania* (Kinetoplastida: Trypanosomatidae) cause the leishmaniases - severe human diseases that may be mutilating or fatal. The *Leishmania donovani* complex (subgenus *Leishmania*) comprising *L. donovani* and *L. infantum* (synonym *L. chagasi*) causes visceral leishmaniasis, which has been responsible for catastrophic epidemics among vulnerable populations of Sudan and the Indian subcontinent and for suburban outbreaks in Brazil. Other members of the genus *Leishmania* cause cutaneous leishmaniasis in both the Old World (*L. major*; *L. tropica*; *L. aethiopica*) and the New World (*L. mexicana*; *L. amazonensis*) or devastating mucocutaneous leishmaniasis (*L. braziliensis* in the New World).

The *Leishmania* life-cycle includes morphologically and physiologically distinct forms in the sand fly vector and in the vertebrate host [1], [2], [3], [4], [5]. Intracellular amastigotes taken up in the sand fly bloodmeal undergo transformation to short procyclic promastigotes with short flagellum. These forms divide and later transform to elongated nectomonads that escape through broken peritrophic matrix [6] and attach to the midgut epithelium to prevent expulsion during defecation of bloodmeal remnants. After defecation, late-stage infections comprise mainly short promastigotes (leptomonads [4]), which migrate anteriorly to the thoracic midgut, and highly motile metacyclic forms infective for the vertebrate host [1], [2], [3], [4], [5].

For many years *Leishmania* parasites have been considered to replicate asexually. No sexual dimorphism has been seen and chromosomes cannot be observed directly because they do not condense during cell division. Although this clonal hypothesis [7] did not entirely rule out the possibility of genetic recombination, such events were believed to be rare and insufficient to disrupt a prevailing clonal population structure. In the absence of genetic exchange it was proposed that recombination in large tandemly repeated gene families was one mechanism by which genetic diversity was sustained [7], [8], [9], [10]. Nevertheless, genetic exchange has been discovered in the related trypanosomatids *Trypanosoma brucei* and *Trypanosoma cruzi*, agents of African and American trypansomiasis, respectively. In both cases these discoveries were initially based on population genetic evidence, followed by formal experimental proof in the laboratory [11], [12], [13], and in both cases genetic exchange events have epidemiological relevance.

During the last two decades, evidence of genetic exchange among natural populations of *Leishmania* has repeatedly emerged in the form of strains that on molecular characterisation appear to be interspecific hybrids [14], [15], [16], [17], [18], [19] or intraspecific hybrids [20], [21]. Genetic exchange was finally demonstrated experimentally in 2009 [22]: 18 hybrid clones of *L. major* were recovered from a natural vector sand fly species (*Phlebotomus duboscqi*) co-infected with two transgenic parental strains. All of the clones inherited a full set of allelic markers from both parents but only one parental maxicircle haplotype. The nuclear genotypes were consistent with a heterozygous first generation progeny. However, 7 of the 18 clones were triploid by analysis of DNA content and the precise mechanism of genetic exchange remains to be determined. A natural progression of such research is to co-infect sand flies with transgenic *Leishmania* carrying two different markers that are fluorescent, in an attempt to visualise the recombination events microscopically [13].

The fact that *Leishmania* can undergo genetic exchange is potentially of profound epidemiological significance. Hybrid offspring might show a strong selective advantage relative to the parental strains. Local abundance of *L. braziliensis/L. peruviana* hybrids was reported from Peru [23] and a putatively recombinant lineage of *L. tropica* has been widely disseminated [24]. Most importantly, hybrids characterised as *L. major/L. infantum* are transmissible by *Phlebotomus papatasi*, a principal vector of *L. major* that is normally refractory to *L. infantum* and *L. donovani* [13], [25], [26], [27].

The localisation and timing of *Leishmania* hybridisation events in the sand fly is as yet unresolved. Transgenic fluorescent trypanosomes have revealed that *T. brucei* undergoes recombination in the salivary glands of the tsetse fly vector [28]. Accordingly, here we have passaged several pairs of red and green transgenic *Leishmania* through appropriate sand fly vector species. We describe the behaviour of the pairs of *L. donovani* strains in competing infections in the sand fly and reveal the presence of unequivocally dual fluorescing hybrid organisms in single *P. perniciosus* and *L. longipalpis* co-infected with *L. donovani* strains LEM 4265 and Gebre-1. Furthermore, high resolution fluorescence microscopy allows provisional definition of the timing and localisation of hybridisation and the morphological stages that are involved.

## Results

### Experimental co-infections

Three pairs of GFP/RFP transfected *L. donovani* strains were used for experimental infections in two sand fly vector species, *P. perniciosus* and *L. longipalpis*: LEM3804 (RFP) and LV9 (GFP), LV9 (GFP) and Gebre-1 (RFP) and LEM4265 (GFP) and Gebre-1 (RFP) (Table 1). Engorged females were dissected at different time intervals PBM and used to study comparative distribution of co-infections, and for attempted recovery of hybrids in non-selective media and double-drug selective slopes, and for FACS analysis.

**Table 1:** Numbers of single and co-infected sand flies examined by fluorescence microscopy.

*L. donovani* strains	Sand fly species	Day 2–3 PBM				Day 4 PBM				Day 7 PBM				Day 9–10 PBM				Total
		Dis	Inf	B	H	Dis	Inf	B	H	Dis	Inf	B	H	Dis	Inf	B	H	Dis
Co-infections																		
LEM3804/LV9	*L. longipalpis*	10	6	6	0	-	-	-	-	12	8	2	0	10	5	3	0	
	*P. perniciosus*	10	10	10	0	-	-	-	-	12	6	5	0	15	10	8	0	
LV9/Gebre-1	*L. longipalpis*	12	12	12	0	-	-	-	-	12	12	4	0	11	7	2	0	
	*P. perniciosus*	10	6	6	0	-	-	-	-	15	10	5	0	15	8	4	0	
LEM4265/Gebre-1	*L. longipalpis*	10	10	10	0	-	-	-	-	15	12	6	0	15	13	5	0	
	*P. perniciosus*	70	56	56	1	40	34	27	0	41	33	21	0	15	10	4	0	
Total	*L. longipalpis*	32	28	28	0	-	-	-	-	39	32	12	0	36	25	10	0	
	*P. perniciosus*	90	72	72	1	40	34	27	0	68	49	31	0	45	28	16	0	
	∑	122	100	100	1	40	34	27	0	107	81	43	0	81	53	26	0	350
Single infections																		
LEM4265	*P. perniciosus*	30	21	-	-	30	23	-	-	38	23	-	-	-	-	-	-	
Gebre-1	*P. perniciosus*	29	23	-	-	30	28	-	-	38	34	-	-	-	-	-	-	
Total	*P. perniciosus*	59	44	-	-	60	51	-	-	76	57	-	-	-	-	-	-	195

PBM, post bloodmeal;

Dis, number of dissected females;

Inf, number of females infected with at least one *Leishmania* strain;

B, number of females infected with both *Leishmania* strains;

H, number of females with hybrid promastigotes seen and photographed under fluorescence microscope;

-, not done.

### Infectivity rates and comparative distribution of co-infections

**Figure 1:**
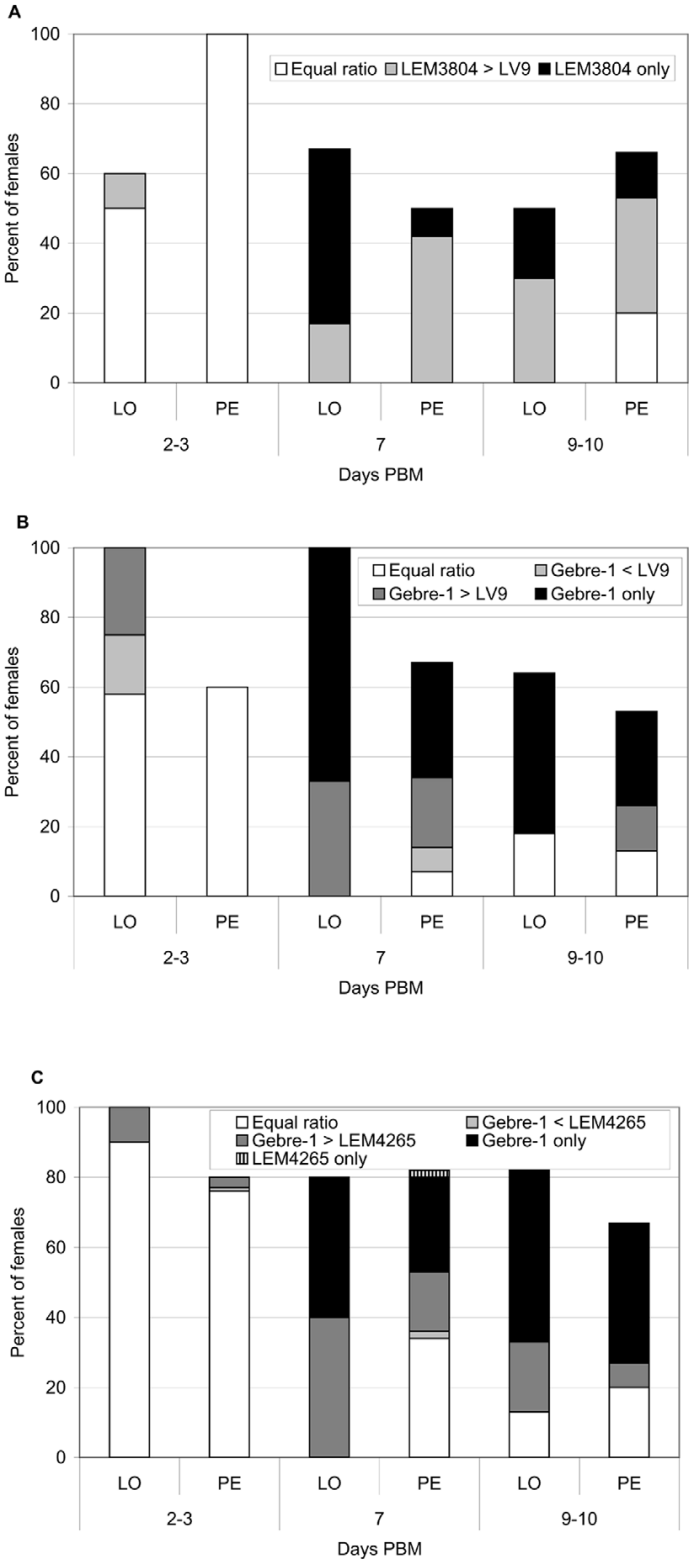
Development of GFP/RFP transfected *Leishmania* strains in sand flies. Infection rates and intensities of co-infections in three combinations of *L. donovani* strains in *L. longipalpis* (LO) and *P. perniciosus* (PE). A, LEM3804 plus LV9: in late stage infections (days 7 and 10) LEM3804 mostly over-grew LV9, in some females LV9 disappeared. B, Gebre-1 plus LV9: Gebre-1 grew better than LV9, in a high proportion of females LV9 was lost during late stage infections. C, Gebre-1 plus LEM4265: in late stage infections Gebre-1 mostly over-grew LEM4265, in a high proportion of females LEM4265 disappeared.

**Figure 2:**
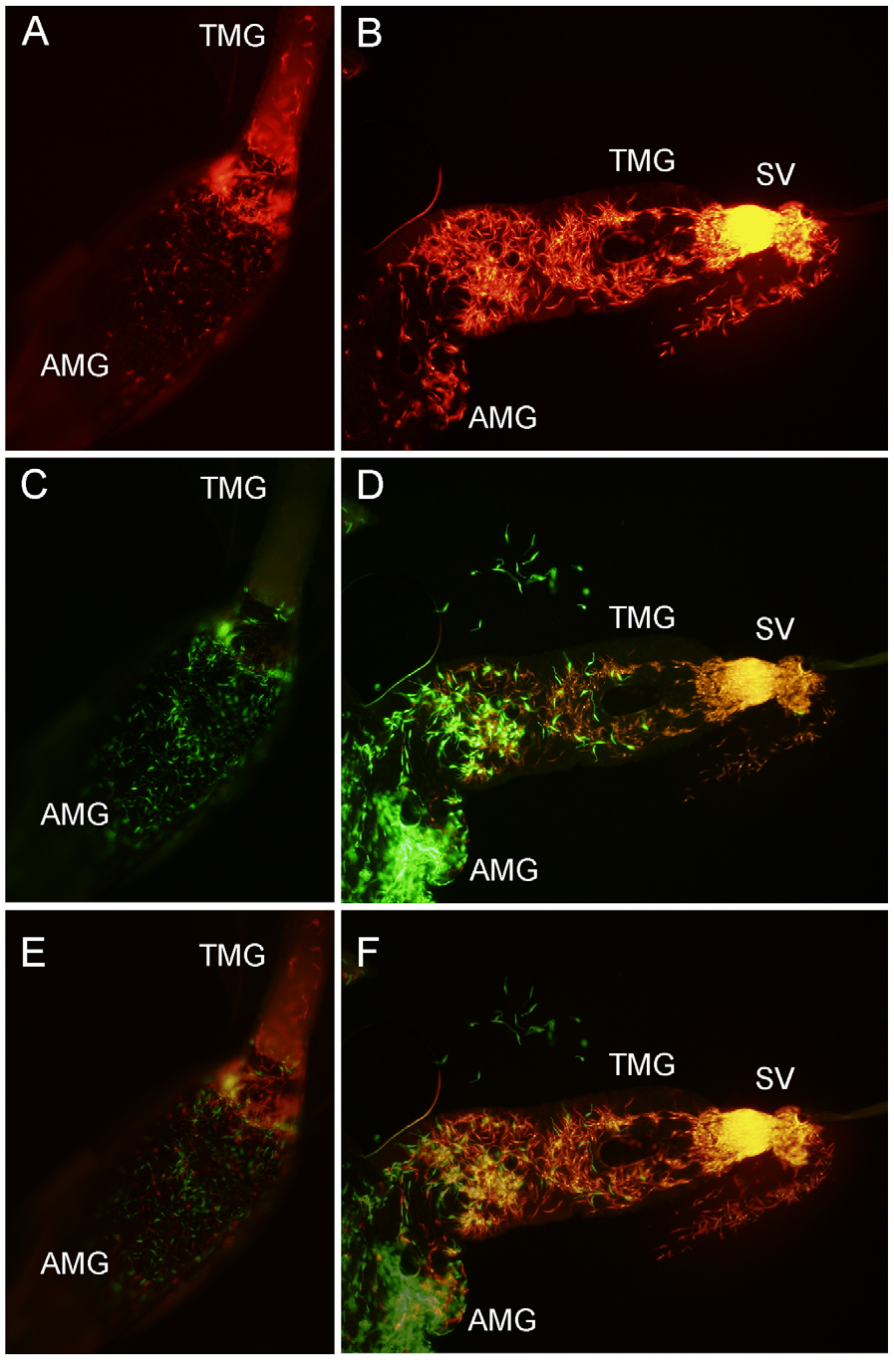
Different location of GFP/RFP transfected *Leishmania* strains in *P. perniciosus.* Images from fluorescent microscopy (Olympus BX51). A, C, E, Early stage of infection (day 3 PBM), parasites of both strains have escaped from the peritrophic matrix to the ectoperitrophic space. LEM3804 parasite (red) starting migration from the abdominal midgut (AMG) into the thoracic part (TMG) while LV9 parasites (green) remained in the abdominal midgut. B, D, F, Late infections (day 7 PBM). Gebre-1 parasites (red) colonizing the stomodeal valve (SV) region while LEM4265 parasites (green) only reached the middle section of the thoracic midgut. A–B, images from red fluorescence, C–D images from green fluorescence and E–F, merged images (x100).

A total of 545 sand fly females were analysed microscopically (Table 1). By day 2–3 PBM the ratio of strains used for co-infections was mostly equal (Fig. 1). But in more established infections (day 7 and 9–10 PBM), LEM3804 prevailed over LV9 and Gebre-1 prevailed over both LV9 and LEM4265 in most specimens of both vector species (Fig. 1). LEM3804 and Gebre-1 infections also reached the cardia and colonized the stomodeal valve (SV) faster than LV9 and LEM4265 (Fig. 2). In these co-infection experiments sand fly females commonly became infected with LEM3804 or Gebre 1 alone, but single infections were absent for LV9 and rare for LEM4265 (Fig. 1).

We also compared development of Gebre-1 and LEM4265 single infections with co-infections, in the same sand fly vector species, *P. perniciosus*. Parasite loads did not differ significantly between single infections and co-infections for either Gebre-1 (N = 191, P = 0.550, χ^2^ = 1.197, d.f.  = 2) or for LEM4265 (N = 192, P = 0.257, χ^2^ = 4.041, d.f.  = 3). Comparisons between single infections and co-infections were performed to determine if the slow colonization of the SV observed with LEM4265 strain was caused by competition with the faster developing Gebre-1 strain. Surprisingly, development of LEM4265 did not differ significantly between the females carrying single infections and co-infections (N = 136, P = 0.477, χ^2^ = 3.509, d.f.  = 4). Parasites of LEM4265 consistently reached the cardia slowly and colonized the SV in a low percentage of females, if infected singly or co-infected with Gebre-1 parasites (Fig. 2).

### Location and morphology of dual fluorescing hybrids found *in situ*

**Figure 3:**
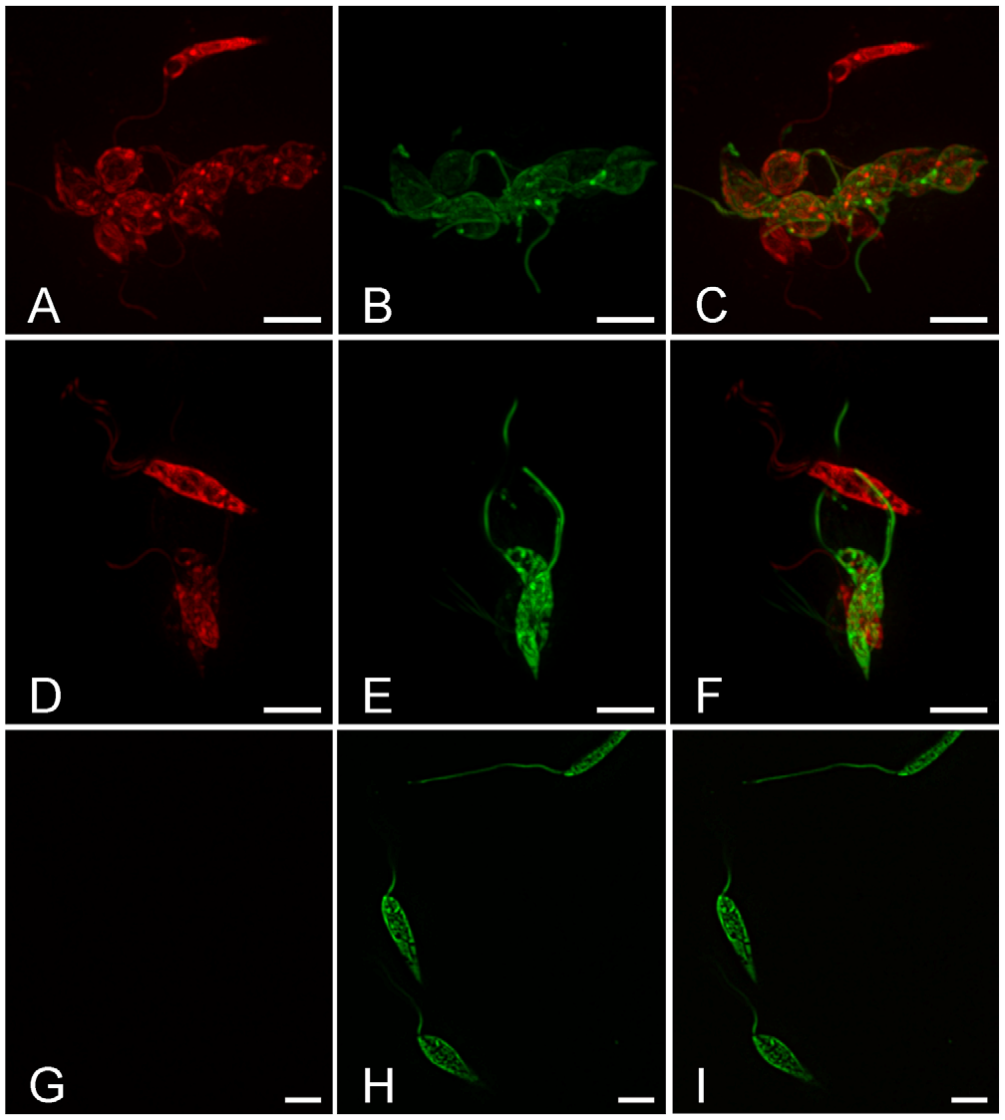
Hybrid promastigotes found in the sand fly midgut. Images from the Olympus Cell^R^ 567 system showing dual fluorescence in *Leishmania* from a *P. perniciosus* female 2 days PBM (A–C). The female was infected with LEM 4265 (GFP transfected) and Gebre-1 (RFP transfected) parental strains. A, B, C, group of 10 hybrids together with four parasites showing red fluorescence only, D–F, group of two hybrids with two red parasites, G–I, GFP controls. A, D, G, images from red fluorescence, B, E, H, images from green fluorescence and C, F, I, merged images, scale bar: 5 µm.

From 350 dissected sand flies that had been allowed to feed on a mixture of two parental GFP/RFP expressing strains, 196 were found to have successfully acquired infection with both strains (Table 1). Hybrid promastigotes clearly and unequivocally showing both red and green fluorescence were found in a single *P. perniciosus* female, co-infected with the LEM 4265 and Gebre-1 parental strains. The hybrid cells were present within the semi-digested bloodmeal in the endoperitrophic space 2 days PBM. Morphologically, hybrid parasites were rounded procyclic promastigotes with average body length 5.3 µm and body width 3.2 µm (n = 10, Fig. 3a–c) or short promastigotes with average body length 9.1 µm, body width 2.3 µm and flagellar length 12.1 µm (n = 2, Fig. 3d–f). All the hybrid cells showed a patched appearance of red and green fluorescence emitting sites (Fig. 3).

### FACS analysis

Numbers of *P. perniciosus* and *L. longipalpis* females co-infected with Gebre-1 (RFP) and LEM 4265 (GFP) examined by flow cytometry are given in Table 2. Although sorting was performed in two sequential steps (see Material and Methods), the resulting events showing both GFP and RFP fluorescence were checked visually by confocal microscopy to distinguish and exclude absolutely FACS sorted sand fly gut remnants with red or green autofluorescence and any sorted pairs of red and green cells from single dual fluorescing hybrid cells, clearly expressing both red and green fluorescence.

**Table 2:** Flow cytometry: numbers of sand flies co-infected with *L. donovani* (Gebre-1, RFP and LEM 4265, GFP) examined post bloodmeal (PBM).

Sand fly species	Time PBM					
	1 day	2 days	3 days	8–9 days*	15–17 days*	Total
*P. perniciosus*	50	50	100	50	143	393
*L. longipalpis*	50	100	35	83	0	268

*In contrast to *P. perniciosus*, shorter longevity of *L. longipalpis* females does not allow later interval of dissections.

**Figure 4:**
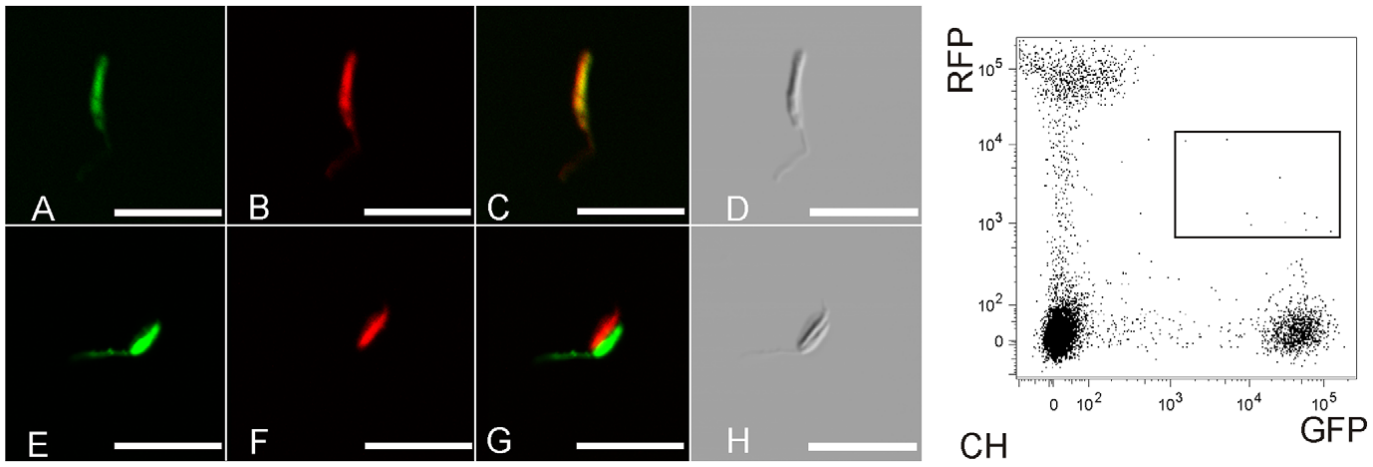
Promastigotes sorted by flow cytometry 2 days PBM. A–H, images from confocal microscopy (Olympus FV1000). A–D, hybrid promastigote, E–H, a doublet of red and green cells. A, E, green fluorescence, B, F, red fluorescence, C, G, RGB images, D, H, phase contrast, scale bar: 10 µm. C, H, Flow cytometry analysis detecting both GFP and RFP fluorescing parasites. A two day PBM sample containing many erythrocytes and non fluorescing particles. Rectangle encompasses the region sorted and screened for hybrids.

From 393 *P. perniciosus* females no putative hybrids were found. From 268 *L. longipalpis*, one *Leishmania* short promastigote, clearly showing colocalized GFP and RFP expression was sorted from the pool of 45 guts dissected at day 2 PBM. Fig. 4 shows clear co-expression of GFP and RFP in this parasite after recovery by FACS. No more hybrid cells co-expressing red and green fluorescence were observed in sand flies dissected after 2 days PBM or during later phases of the *Leishmania* infection. The frequency of hybridisation in *L. longipalpis* (based on phenotype) was estimated to be 4.8×10^−5^ or less.

### Recovery of hybrids

Initially, when both selective drugs were incorporated into the overlay, 25 isolates were recovered from 121 dissected females. However, when the selective antibiotics were incorporated into both components of the cultivation medium, the blood agar and the overlay, no further isolates were recovered from 89 experimental co-infections. Nor did the 25 positive isolates sustain growth in this highly selective medium. Furthermore, none of the 25 isolates contained both neomycin and hygromycin drug resistance markers after PCR amplification. In addition, 157 isolation attempts from the sand fly co-infections were made into non-selective medium. Of these 70 produced viable cultures but growth was not sustained for more than 1 or 2 sub-cultures in double drug selective medium. Thus, although putative hybrids were clearly seen in sand flies and a single putative hybrid was recovered by FACS, viable hybrids could not be grown in vitro.

## Discussion

*Leishmania* have, controversially, been considered to be propagated clonally. However, accumulating evidence from genotyping of natural populations of both Old World and New World *Leishmania* has revealed the presence of putative hybrids between several combinations of described species and between strains within species. Finally, in 2009, the landmark experiments of Akopyants et al. [22] proved that two strains of *L. major* carrying different drug resistance markers could undergo genetic exchange, and that this process occurred in the sand fly vector, with an approximate frequency of 2.5×10^−5^.

We have described a quite extensive series of experiments passaging three pairs of transgenic drug resistant GFP or RFP labelled *L. donovani* strains through their natural sand fly vectors *P. perniciosus* and *L. longipalpis* and dissecting at intervals PBM. Gut contents were examined by fluorescence microscopy and by FACS analysis and also subjected to double drug selections in an attempt to recover viable hybrids.

In experiments aimed at examining *L. donovani* behaviour in *P. perniciosus* and *L. longipalpis* we obtained 196 successful co-infections from 350 sand fly females given bloodmeals containing pairs of *L. donovani* strains (Table 1). Multiple hybrids were seen in a single *P. perniciosus* co-infected with the strains LEM4265 and Gebre 1, checked *in situ*. Retrieval of a single cell unequivocally expressing both fluorescent reporters was achieved also by FACS analysis from the pool of *L. longipalpis* co-infected with the same combination of parental strains. This demonstrated the immense value of FACS for detecting and sorting hybrid progeny.

In these co-infections, the strains involved did not appear to behave competitively. Strains displayed the same broad level of distribution of infection in single and co-infected flies. We did not recover sustained growth of viable hybrid progeny in non selective media or through rigorous double drug selection. However, the importance of this work is that the fluorescence did allow us to visualise the distribution and course of the infection in the sand flies with great clarity, to see hybrid organisms displaying both red and green fluorescence and to make several key, novel observations. This demonstrates that the *L. donovani* complex almost certainly has the capacity for genetic exchange within its natural sand fly vector species, and confirms the validity of this experimental approach.

Furthermore, we made observations indicating that hybridisation events probably occur in the early phase of bloodmeal digestion, 2 days PBM, when all parasites are located in the endoperitrophic space within the bloodmeal surrounded by peritrophic matrix. We obtained spectacular high resolution images of the promastigote morphological stages involved, as illustrate in Figures 3 and 4. However, hybrids were not seen in long term sand fly infections and proved to be non-viable in the culture conditions used here and as a result we were unable to characterise hybrid progeny in detail by molecular methods.

These data from microscopic examinations and FACS experiments suggest that similarly low frequency of sexual events occurred in both tested sand fly species. The low frequency of hybrid occurrence has several possible explanations. The strains used had been maintained in the laboratory for prolonged periods. Some genotyping data were available but genetic compatibilities are not known. Furthermore, sand flies were infected with promastigotes derived from 4 day old cultures, not with amastigotes within macrophages, and bloodmeals were artificially comprised. However, in a previous study *L. major* hybrids [22] were achieved using the same mode of sand fly infection with strains maintained long-term in culture, yielding 12 positive double drug resistant isolates from only 102 dissected sand flies.

Our findings suggest that there may be a species-specific difference among *Leishmania* in the capacity for sexual reproduction. Rarity of genetic exchange in our experiments may reflect natural situations where there is minimal chance of hybrid clones arising and being more viable than parental genotypes. However, hybridisation events when they occur may fail to yield progeny that are viable in the long term or progeny that have a selective advantage and allow the hybrids to out-compete parental populations. Genetic exchange may infrequently break a prevailing pattern of clonal population structure [7].

Nevertheless, the success of hybridization and its epidemiological importance in *Leishmania* is unquestionable from genetic analysis of natural populations, and in particular from experimental demonstration of the vigour and transmissibility of hybrids described as *L. major*/*L. infantum* [25]. Future experimental crosses with transgenic *Leishmania* should a) be based on putative parental and hybrid strains recently isolated in endemic regions from sympatric populations b) probably employ strains predetermined to have similar growth rates and intensities in sand flies c) incorporate sequential as well as mixed infections d) include genetically similar strains to allow that genetic exchange might frequently involve closely related populations. We have observed that hybrid formation can occur between *L. donovani* strains in the natural vector sand fly species, and that the cell types involved are short (procyclic) promastigotes as early as 2 days PBM. We have also shown that fluorescence microscopy with FACS analysis and sorting of individual cells provides a strong combined approach for visualization and recovery of hybrid progeny.

## Materials and Methods

### *Leishmania* strains

Four *Leishmania donovani* strains were used in experimental crosses: LEM3804 (MCAN/SD/99/LEM3804), LV9 (MHOM/ET/67/L82/LV9), Gebre-1 (MHOM/ET/72/GEBRE1) and LEM4265 (MHOM/SD/2001/AHSAF2). Strains originate from Sudan (LEM 3804, LEM 4265) or Ethiopia (LV9, GEBRE1) and could be distinguished, in our laboratory, by SNP differences in four housekeeping genes. Preliminary experiments confirmed that all these strains were able to develop in sand flies and establish heavy late-stage infections with colonization of the stomodeal valve. All the strains were maintained as stabilates and at 23°C either in liquid alpha-MEM medium (minimum essential media, Invitrogen) supplemented with 20% heat inactivated FCS or in blood agar base over-laid with alpha-MEM. G418 and hygromycin were added to final concentrations of 150 µg/ml when double drug selection was applied, as described below.

### Preparation of transgenic fluorescent *Leishmania* strains

Parental lines (LEM3804, LV9, Gebre-1, LEM4265) of transgenic fluorescent *Leishmania* were produced using the LEXSY expression system (Jena Bioscience) and the expression vectors pF4X1.4neo and pF4X1.4hyg incorporating eGFP and RFP, respectively, as fluorescent markers. This vector contains optimised untranslated regions flanking the target gene insertion site and splicing signals for post-transcriptional mRNA processing. Insertion of eGFP and RFP was verified by vector cleavage using restriction enzymes (*Nco*I and *Not*I) to confirm replacement of the 0.7kb stuffer fragment with the appropriate fluorescent protein cassette. The expression vectors were initially constructed in *E. coli* and introduced into *Leishmania* by electroporation using d = 4 mm cuvettes and two pulses at 1500V, 25uF [29]. Expression of the target genes in *Leishmania* followed integration of the expression cassette into the chromosomal 18S rRNA locus (SSU). Integration was confirmed by diagnostic PCR amplification from genomic DNA, with one primer hybridising within the expression cassette and one to a SSU sequence not present in the plasmid. Expression of eGFP and RFP, which is dependent on successful integration, was confirmed by fluorescence microscopy and transgenic *Leishmania* clones showing stable fluorescence were selected for the crossing experiments. Transgenic cell lines retained vigorous growth in vitro.

### Experimental infection of sand flies

Separate colonies of *P. perniciosus* and *L. longipalpis,* proven vectors of *L. infantum*, part of the *L. donovani* complex, were maintained at 26°C, on a 14-h light/10-h dark photoperiod [30] and fed on a 50% sucrose solution. Sand fly females were infected by feeding through a chick-skin membrane on heat inactivated rabbit blood (purchased from Bioveta, Ivanovice na Hane, Czech Rep.) containing a mixture of two parental strains at 10^6^ promastigotes/ml, derived from four-day old α-MEM or blood agar cultures. Engorged sand flies were maintained in the same conditions as the colony and dissected at various time intervals PBM (Table 1) either for microscopic observation or for isolation of *Leishmania*.

### Recovery of live *Leishmania* from sand flies

To recover live *Leishmania,* sand flies were dissected in sterile saline and gut contents were inoculated into blood agar slopes overlaid with alpha-MEM medium supplemented with 20% heat inactivated FCS, fluorocytosine (100 ug/ml) and gentamicin (100 ug/ml). Selective antibiotics (150 ug/ml hygromycin, 150 ug/ml G418) were applied either in the liquid overlay or in both the overlay and agar base of the primary cultures. However, some primary cultures were inoculated without the addition of selective antibiotics, and double drug selection was only applied when recovered *Leishmania* were sub-cultured. Cultures were examined weekly by microscopy for the presence of *Leishmania* and if positive the promastigotes were cryopreserved in liquid nitrogen for further characterisation of the recovered organisms.

### Dissection of sand flies and microscopy

Dissected sand fly guts were fixed *in situ* on the slide with 1% paraformaldehyde and examined by microscopy. Levels of *Leishmania* infection were graded as reported previously [31] as light (<100 parasites/gut), moderate (100–1000 parasites/gut) and heavy (>1000 parasites/gut). The course of infection and location of *Leishmania* infection, including individual parasites, in the sand fly digestive tract were determined and recorded photographically with an Olympus BX51 fluorescent microscope and Olympus camera. Putative hybrids expressing both red and green fluorescence were detected by merging images from red and green fluorescence filters using Adobe Photoshop 7.0.1. In addition, guts were also very carefully examined with an Olympus Cell^R^ system which provides multi-fluorescence imaging and allows direct scrutiny of hybrid parasites emitting both green and red fluorescence. Putative hybrids sorted by flow cytometer were checked and single cells scrutinised and photographed with confocal fluorescence microscopy on an Olympus FV1000.

### Preparation of *Leishmania* clones

*Leishmania* recovered in selective media were cloned by plating on solid media using the methodology described by [32]. Ten clonal colonies were selected from each plate and transferred to non selective media (alpha-MEM supplemented with 20% heat inactivated FCS). Following DNA extraction (Qiagen) the presence of the expression cassette, containing resistance and fluorescent markers, was confirmed by PCR amplification (30 cycles of 30 sec at 94°C, 1 min at 53°C, and 2 min at 72°C), with the primers A3804 (CCGATGGCTGTGTAGAAGTACTCG) and F3002 (CTGCAGGTTCACCTACAGCTAC). Integration of the expression cassette into the ssu locus resulted in a characteristic 1.8 kbp fragment.

### FACS analysis

At different time intervals PBM (Table 2), guts of infected sand flies were dissected into a small volume of the cultivation medium (35–50 guts in 15 µl), filtered using a 30 µm filter (Partec) into PBS buffer and analyzed with a LSRII flow cytometer (BD Biosciences). Numbers of GFP positive events (excitation with the 488 nm laser, emission detection at 530/30 nm), RFP positive events (excitation with the 561 nm laser, emission detection at 585/15 nm) and putative hybrid cells, i.e. events simultaneously emitting at both GFP and RFP wavelengths, were determined. The cytometer was calibrated using both positive controls (GFP transfected *Leishmania* line, RFP transfected *Leishmania* line) and a negative control (wild type *Leishmania* strain).

Putative hybrid cells were sorted with FACSVantage SE cell sorter (BD Biosciences) with the laser emitting at 488 nm wavelengths and detection of emission at 530/30 nm (GFP positive events) and 585/42 nm (RFP positive events). Sorting was performed in two steps: in the first phase all GFP positive events were separated, secondly a further sorting was performed on the GFP separated parasites. Events showing double positive emission were collected into a glycerol drop containing 1% formaldehyde and immediately checked visually on the Olympus FV1000, for single cells co-expressing red and green fluorescence (See Results, FACS analysis, above)..

### Ethics Statement

This study does not include experiments on animals or humans.
